# Underlying microangiopathy and functional outcome of simultaneous multiple intracerebral hemorrhage

**DOI:** 10.3389/fnagi.2022.1000573

**Published:** 2022-11-08

**Authors:** Jiawen Li, Dan Shen, Yanli Zhou, Yujia Jin, Luhang Jin, Xianghua Ye, Lusha Tong, Feng Gao

**Affiliations:** Department of Neurology, The Second Affiliated Hospital, Zhejiang University School of Medicine, Hangzhou, Zhejiang, China

**Keywords:** intracranial hemorrhages, magnetic resonance imaging, cerebral small vessel diseases, prognosis, stroke

## Abstract

**Objective:**

To identify the predominant type of cerebral small vessel disease (SVD) and outcomes in patients with simultaneous multiple intracerebral hemorrhages (SMICH).

**Methods:**

Consecutive patients with intracerebral hemorrhage (ICH) from a single-center prospective cohort were retrospectively reviewed. Presumed etiology was classified according to the SMASH-U criteria. Demographics, clinical and laboratory variables, and neuroimaging data were compared between patients with primary SMICH and those with single ICH. Functional outcomes were assessed using the modified Rankin scale 90 days after ICH.

**Results:**

Of the 598 enrolled patients, 37 (6.2%) met the criteria for SMICH. Risk factors for SMICH included a high burden of deep cerebral microbleeds (CMBs) (odds ratio [OR] 1.06, 95% confidence interval [CI], 1.00–1.12; *p* = 0.040), white matter hyperintensity scores (OR 1.27, 95% CI 1.04–1.57; *p* = 0.021), history of ICH (OR 3.38, 95% CI 1.31–8.05; *p* = 0.008), and low serum magnesium levels (OR 0.01, 95% CI 0.00–0.25; *p* = 0.007). Based on the SMASH-U classification, 15(40.5%) SMICH were classified as hypertension, whereas 17 (45.9%) as undetermined-etiology. To further explore the potential microangiopathy underlying undetermined-SMICH, these patients with undetermined-etiology were compared to those with cerebral amyloid angiopathy-ICH, and were associated with a higher burden of deep CMBs but less severe centrum semiovale enlarged perivascular spaces. Likewise, compared with hypertension-ICH patients, those with undetermined SMICH were consistently associated with a higher deep CMB counts. Moreover, multivariate analysis revealed that SMICH was independently associated with poor outcomes (OR 2.23, 95%CI 1.03–4.76; *p* = 0.038).

**Conclusion:**

Our results suggest that most patients with primary SMICH harbor hypertensive-SVD as principal angiopathy. Patients with SMICH are at a high risk of poor outcomes. (ClinicalTrials.gov Identifier: NCT 04803292).

## Introduction

Simultaneous multiple intracerebral hemorrhages (SMICH) are characterized by bleedings within different arterial territories. SMICH represents 3.6–5.9% of all hemorrhagic stroke, (Stemer et al., 2010; Yeh et al., 2014; Chen et al., 2016; Wu et al., 2017) however, it is associated with a higher morbidity and mortality rate compared with patients with single hematoma (Yen et al., 2005; Chen et al., 2016). Hypertensive angiopathy and cerebral amyloid angiopathy(CAA) are the major causes of primary intracerebral hemorrhage(ICH; Qureshi et al., 2001). Generally, hypertension leads to arteriolosclerosis in the deep perforating arteries while CAA is characterized by amyloid deposition in cortical/leptomeningeal microvessels (Pantoni, 2010; Viswanathan and Greenberg, 2011; Charidimou et al., 2012; Samarasekera et al., 2012). Based on pathology studies, Thal et al. (2003) proposed that CAA pathology could extend to the basal ganglia and brain stem vessels, and hypertension-related changes also cause lobar microbleeds (Broderick et al., 1993; Vernooij et al., 2008; Kim et al., 2016; Rodrigues et al., 2018). Whether patients with SMICH had advanced hypertensive angiopathy or CAA or a combination of both remained unclear. Figuring out the underlying angiopathy is clinically crucial for guiding treatment decisions and planning secondary preventive measure.

Magnetic resonance imaging (MRI) technology has emerged as the most useful noninvasive method for diagnosing angiopathies associated with primary ICH. Enlarged perivascular spaces (EPVS) in the centrum semiovale (CSO) and exclusively cortical cerebral microbleeds (CMBs) are consistently associated with CAA. In contrast, EPVS in the basal ganglia (BG) and deep CMBs presumably account for hypertensive angiopathy (Greenberg et al., 2009; Charidimou et al., 2013, 2017; van Veluw et al., 2022). Previous studies noted that there was a higher burden of total CMBs among SMICH patients; (Stemer et al., 2010; Chen et al., 2016) however, the number of CMBs stratified by different locations (deep vs. lobar) had not been evaluated, and the phenotypes of other cerebral small vessel disease (SVD) MRI markers remained unclear.

This study aimed to describe the detailed imaging profiles and 90-day functional outcomes of patients with SMICH. We investigated: (a) the prevalence of primary SMICH, (b) the principle angiopathy underlying SMICH, (c) the relationship between SMICH and the 90-day poor outcome of ICH.

## Materials and methods

### Patient recruitment

**Figure 1:**
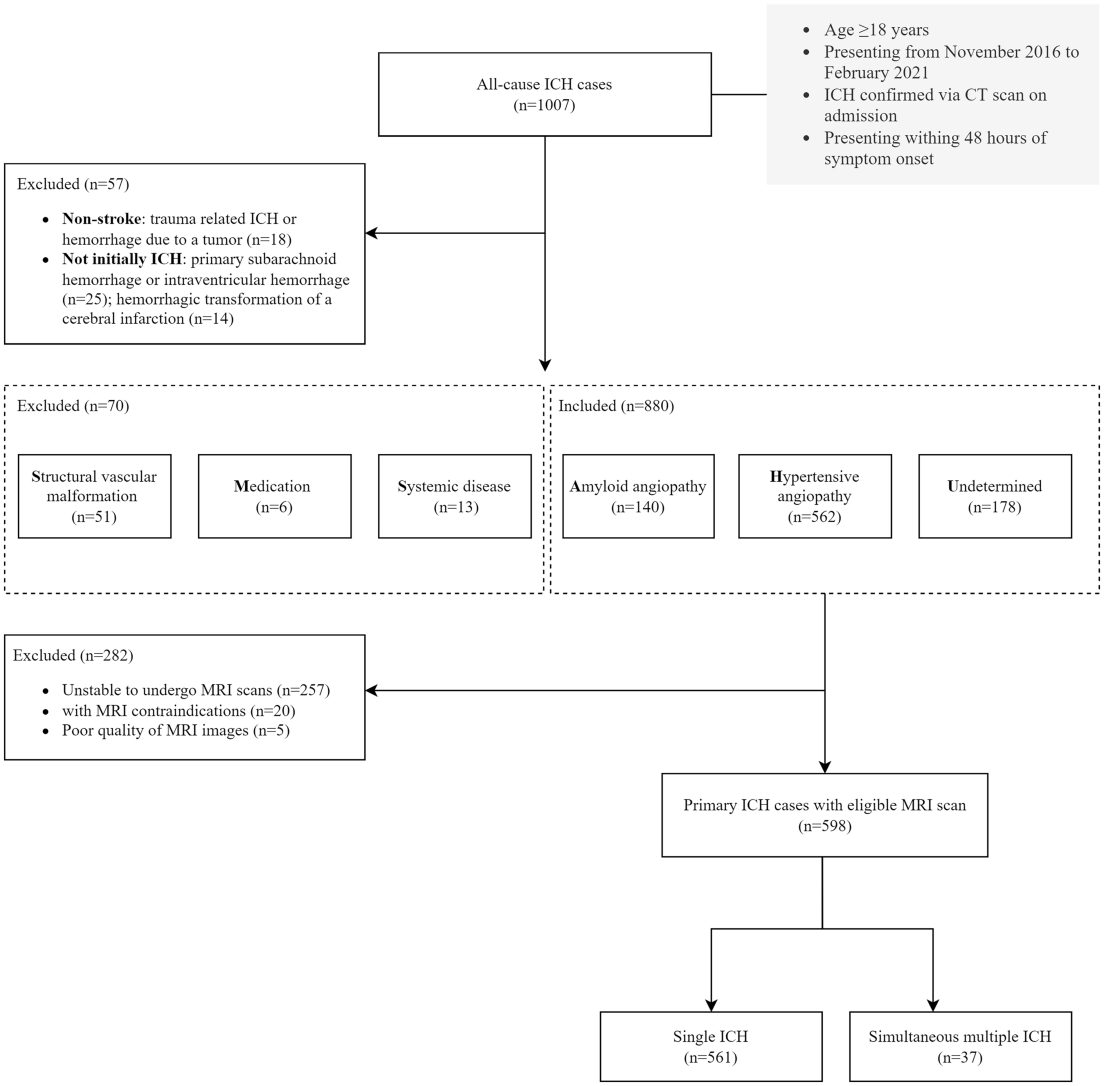
Flow chart of the study population.

We retrospectively reviewed our data collected from a longitudinal ICH cohort study conducted at the Second Affiliated Hospital of Zhejiang University. The study included patients with ICH admitted to the Department of Neurology between November 2016 and February 2021. Eligibility criteria included admission within 48 h after ICH ictus and age ≥ 18 years. The ICH diagnosis was confirmed on admission *via* computed tomographic (CT) scan. Traumatic ICH, hemorrhage due to brain tumor, and those were not initially ICH (i.e., primary subarachnoid hemorrhage, primary intraventricular ICH, and hemorrhagic conversion of cerebral infarction) were diagnosed in routine clinical examination and excluded. We classified ICH etiology by using SMASH-U as structural vascular lesions (S), medication (M), amyloid angiopathy (A), systemic disease (S), hypertension (H), or undetermined (U; Meretoja et al., 2012). Specifically, all the patients enrolled had CT angiography or magnetic resonance angiography, or if necessarily, digital subtraction angiography, to find out underlying structural vascular pathology at the site of the ICH; Medication-related ICH was classified as a patient on warfarin with international normalized ratio ≥ 2.0, novel oral anticoagulants within 3 days, full-dose heparin, or systemic thrombolysis of noncerebral thrombosis. Systemic or other causes of ICH include liver cirrhosis, systemic coagulopathy (platelet count≤50 × 10^9^/L), cerebral venous sinus, endocarditis, and cerebral vasculitis without infarction. Cerebral amyloid angiopathy(CAA) was defined by patients aged ≥55 with lobar, cortical, or cortico-subcortical hematoma. Hypertensive angiopathy was considered in patients with hemorrhage restricted to deep or infratentorial regions (the basal ganglia, thalamus, or brainstem) with pre-ICH hypertension. This study included patients with CAA, hypertensive angiopathy and an undetermined etiology as primary ICH cases. Finally, only those with eligible brain MRI scans during hospitalization were included in our analysis (Figure 1).

SMICH was defined as at least two non-contiguous acute hematomas in different parenchymal territories Yen et al. (2005). Li JW and Shen D individually reviewed initial CT images to determine the presence of SMICH. In case of disagreement, Tong LS was consulted for a final decision. In SMICH, all hematomas were used for location and etiologic determination. Patients who did not fulfill the above criteria were classified as undetermined (patients with co-existing lobar and deep/infratentorial ICH, strictly lobar ICH but <55 years, or with strictly deep/infratentorial ICH but without pre-ICH hypertension).

### Ethical standards

This research was conducted in accordance with the Declaration of Helsinki and was approved by the Human Ethics Committee of the Second Affiliated Hospital of Zhejiang University, School of Medicine. Informed consent was obtained from all participants or their guardians.

### Clinical data

Data regarding demographics (age and sex), medical history (hypertension, diabetes mellitus, prior ICH, current medications, and tobacco use), baseline blood pressure, Glasgow Coma Scale (GCS) score, and National Institutes of Health Stroke Scale (NIHSS) score were systematically evaluated and recorded on admission by a senior certified neurologist (Zhou YL). We prespecified tobacco use as a current smoker or having a recent (<2 years) smoking history over 20 cumulative pack-years Sembill et al. (2018). Baseline laboratory data were retrieved from medical records, including serum calcium and magnesium levels, international normalized ratio, activated partial thromboplastin time (APTT), and creatine. The presence of left ventricular hypertrophy, as a biomarker of hypertension, on transthoracic echocardiography was also recorded when available.

## Neuroimaging

### Hematoma assessment

The CT scan protocol has been previously described (Xu et al., 2019). Two independent investigators (Jin YJ and Jin LH) blinded to clinical and outcome data ascertained hemorrhage locations and hematoma volumes. Hematoma on CT was classified as lobar if it involved only cortical or subcortical junction regions. The location was classified as deep when hematoma appeared in the basal ganglia or thalamus, and infratentorial meant location within the brainstem or cerebellum, separately. The presence of intraventricular extension was also recorded. Hematoma volume was measured with a semiautomated planimetric method using ITK-SNAP software (University of Pennsylvania, Philadelphia, United States)^1^. Intraventricular hemorrhage volume was not included in the hematoma volume. In each patient with SMICH, the total hematoma volume was calculated by summing all the lesions.

### MRI acquisition and analysis

The parameters of each MRI sequence had been described in detail (Xu et al., 2019; Li et al., 2020; Ye et al., 2021). Briefly, MRI images were obtained using a 1.5-T or 3.0-T MR scanner and included whole brain T1 weighted, T2 weighted, fluid-attenuated inversion recovery (FLAIR), and susceptibility-weighted imaging (SWI).

Neuroimaging markers of cerebral small vessel disease were evaluated according to STRIVE (Standards for Reporting Vascular Changes on Neuroimaging) consensus criteria (Wardlaw et al., 2013) Cerebral microbleeds (CMB) were visualized as round or oval focal areas of hypointense foci on SWI sequences with a maximum diameter of 10 mm. In this study, the lobar, deep, and total CMB number were separately counted according to the previously validated Microbleed Anatomical Rating Scale. White matter hyperintensity (WMH) was rated visually on axial FLAIR images using the 4-point Fazekas’ rating scale. The total WMH score was the addition of the scores for periventricular white matter and deep white matter hyperintensities (Fazekas et al., 1987). Enlarged perivascular spaces (EPVS) were rated on axial T2 weighted imaging in the basal ganglia (BG) and centrum semiovale (CSO). We prespecified a dichotomized classification of EPVS degree as high (score > 20) or low (score ≤ 20; Charidimou et al., 2013, 2017). The presence or absence of cortical superficial siderosis (cSS) was visually assessed according to the current consensus criteria (Charidimou et al., 2015). All MRI analyses were assessed by trained investigators (Jin YJ and Jin LH) blinded to all clinical and outcome information.

### Outcome measures

We assessed functional outcomes on day 90 using the mRS *via* telephone calls with patients or their caregivers after enrollment until June 30, 2021. The mRS score was classified as functional dependence (mRS grades 3–6) or functional independence (0–2) for outcome evaluation.

### Statistical analyses

The continuous variables had a non-normal distribution according to the Shapiro–Wilk test; therefore, data was expressed as median and interquartile ranges and analyzed using the Mann–Whitney *U* test. Categorical variables were expressed as *n* (%) and compared between groups using Pearson’s *χ*^2^ or Fisher’s exact test, as appropriate. Demographic, clinical and neuroimaging differences were explored in the univariate analysis between patients with (i) SMICH and single ICH, and (ii) undetermined-SMICH vs. all CAA-ICH patients and those with HTN-ICH (i.e., including single ICH and SMICH). To identify the independent factors associated with SMICH, a multivariate logistic regression analysis was performed using the following potential effectors with *p*-values < 0.1 in the univariate comparisons or were relevant based on prior reports: age, male sex, previous ICH, APTT, serum magnesium levels, and CSVD imaging markers (lobar CMB counts, deep CMB counts, total WMH score, and BG EPVS). The final model was built using a backward elimination strategy (*p* < 0.05 to retain). There were 17 undetermined-SMICH cases; to prevent overfitting of the models, hence, we built two models including three variables at a time to identify the independent factors associated with undetermined SMICH vs. CAA-ICH and HTN-ICH: the first model included age, deep CMB counts, and BG EPVS, and the second model included age, deep CMB counts, and CSO EPVS. The association with the 90-day functional independence was assessed using a multivariate logistic regression model adjusted for age, male sex, baseline GCS, previous ICH, ICH volume, presence of IVH, and infratentorial location. For sensitive analysis, matching 1:3 on the propensity score was performed using a nearest neighbor-matching algorithm with a maximum caliper of 0.2 of the propensity score on baseline demographics (age and male sex), co-morbidities(hypertension, diabetes mellitus, previous ICH), medication use (antiplatelet agent, anticoagulation, statins), ICH volume, and ICH etiology. To validate the results from the logistic regression model, the primary analysis was repeated with matched controls. We checked for multicollinearity of each variable using a Variation Inflation Factor (VIF) test, assuming VIF > 2 as benchmark for collinearity. All the analyses were performed using R 4.1.0.^2^ A 2-sided *p* value <0.05 was considered significant.

## Results

After applying the pre-specified inclusion and exclusion criteria to 1,007 consecutive ICH participants (Figure 1), 598 primary ICH participants with MRI sequences (median age 62 years, 52–70 years, 65.6% male sex) were enrolled in the final analysis set. See Supplementary Table 1 for differences between the patients with and without MRI. The clinical and neuroimaging characteristics of all the included participants are summarized in Table 1.

**Table 1:** Baseline clinical, laboratory, and radiological variables of all patients and patients grouped by simultaneous multiple intracerebral hemorrhage.

	Overall (n = 598)	Single ICH (n = 561)	SMICH (n = 37)	Value of *p*
**Age at enrollment, *y***	62 (52–70)	61 (51–69)	66 (55–75)	0.055
**Male sex**	392 (65.6%)	367 (65.4%)	25 (67.6%)	0.790
**Onset to emergency, *h***	6.0 (3.0–18.0)	6.0 (3.0–16.0)	7.0 (3.0–24.0)	0.214
**GCS**	15 (13–15)	15 (13–15)	15 (13–15)	0.442
**NIHSS**	4 (2–10)	4 (2–10)	8 (3–10)	0.015*
**Hypertension**	453 (75.8%)	424 (75.6%)	29 (78.4%)	0.700
**Diabetes mellitus**	99 (16.6%)	93 (16.6%)	6 (16.2%)	0.626
**Previous ICH**	45 (7.5%)	36 (6.4%)	9 (24.3%)	<0.001**
**Antiplatelet**	51 (8.5%)	46 (8.2%)	5 (13.5%)	0.232
**Anticoagulation**	2 (0.3%)	2 (0.4%)	0 (0.0%)	1.000
**Statin**	32 (5.4%)	30 (5.4%)	2 (5.4%)	1.000
**Tobacco use**	207 (34.6%)	192 (34.2%)	15 (40.5%)	0.434
**Serum Calcium, mmol/L**	2.27 (2.19–2.35)	2.27 (2.19–2.35)	2.28 (2.20–2.34)	0.794
**Serum Magnesium, mmol/L**	0.86 (0.81–0.91)	0.86 (0.81–0.92)	0.82 (0.77–0.88)	0.007*
**APTT, s**	34.30 (31.72–36.90)	34.20 (31.70–36.80)	35.40 (33.80–38.20)	0.040*
**INR**	1.00 (0.96–1.05)	1.00 (0.96–1.05)	1.00 (0.99–1.07)	0.271
**Creatine, umol/L**	61 (51–72)	61 (51–72)	60 (48–76)	0.766
**ICH volume, mL**	9.0 (3.2–17.7)	8.8 (3.–17.3)	16.0 (6.6–30.9)	0.006*
**Presence of IVH**	184 (30.8%)	167 (29.8%)	17 (46.0%)	0.039*
***ICH etiology***				
**Amyloid angiopathy**	85 (14.2%)	80 (14.3%)	5 (13.5%)	0.900
**Hypertensive angiopathy**	382 (63.9%)	367 (65.4%)	15 (40.5%)	0.002*
**Undetermined**	131 (21.9%)	114 (20.3%)	17 (45.9%)	<0.001**
**Lobar CMB count**	0 (0–2)	0 (0–2)	2 (0–7)	<0.001**
**Deep CMB count**	1 (0–3)	1 (0–3)	5 (1–7)	<0.001**
**Total WMH burden(Fazekas score)**	2 (1–4)	2 (1–4)	5 (2–6)	<0.001**
**CSO EPVS high degree (score > 20)**	68 (11.4%)	63 (11.2%)	5 (13.5%)	0.598
**BG EPVS high degree (score > 20)**	101 (16.9%)	89 (15.9%)	12 (32.4%)	0.009*
**Lacunes ≥ 1**	382 (63.9%)	354 (63.1%)	28 (75.7%)	0.123
**Presence of cSS**	105 (17.6%)	97 (17.3%)	8 (21.6%)	0.502

Data are median (interquartile range) or n (%). Abbreviations: SMICH, simultaneous intracerebral hemorrhages; GCS, glasgow coma scale; NIHSS, national institute of health stroke scale; SBP, systolic blood pressure; DBP, diastolic blood pressure; ICH, intracerebral hemorrhage; APTT, activated partial thromboplastin time; INR, international normalized ratio; IVH, intraventricular hemorrhage; CMB, cerebral microbleed; WMH, white matter hyperintensities; CSO, centrum semiovale; BG, basal ganglia; EPVS, enlarged perivascular spaces; cSS, cortical superficial siderosis. *p < 0.05. **p < 0.001.

**Figure 2:**
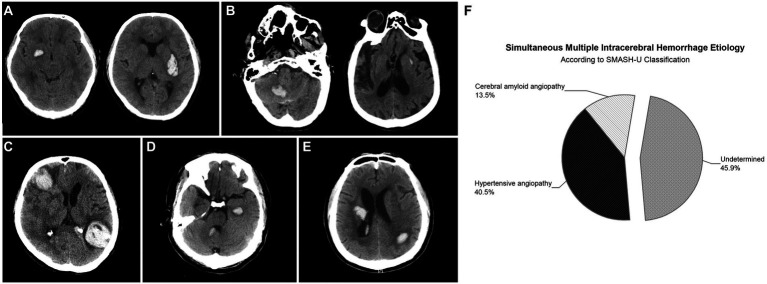
Representative examples of patients with simultaneous multiple intracerebral hemorrhage (SMICH). According to SMASH-U classification, patients who had ICH restricted to deep **(A)** or infratentorial **(B)** regions with pre-ICH hypertension were classified as hypertensive angiopathy; Cerebral amyloid angiopathy (CAA) was defined by patients aged ≥55 with lobar hematoma **(C)**. Patients with co-existing lobar and deep/infratentorial ICH were classified as undetermined-SMICH **(D,E)**. Cerebral amyloid angiopathy (*n* = 5 [13.5%]) and hypertension (*n* = 15 [40.5%]) were common classifications, whereas 17 (45.9%) patients with primary SMICH had an undetermined etiology **(F)**.

SMICH was identified in 37 (6.2%) participants according to the criteria and the representative images were presented in Figure 2. There were 76 discrete ICH lesions in the SMICH group: 35 patients (94.6%) had two hematomas, while the remaining had three. The topographic distribution was as follows: 17 lenticular nuclei, 14 thalami, 7 internal capsules, 4 caudate nuclei, 10 temporal lobes, 6 frontal lobes, 6 occipital lobes, 4 parietal lobes, 6 cerebella, and 2 pontes. Based on MRI examination, no new hematoma occurred compared to the baseline image.

According to the SMASH-U classification, amyloid angiopathy (*n* = 5 [13.5%]) and hypertension (*n* = 15 [40.5%]) were the common etiologies, whereas 17 (45.9%) patients with primary SMICH had an undetermined etiology.

### Baseline characteristics associated with SMICH

On univariate analysis, comparing SMICH to single ICH, patients with SMICH were similar in sex distribution, tended to be older (*p* = 0.055), but they were more likely to have ICH history, IVH, higher NIHSS score, larger hematoma volumes, longer APTT, and lower serum magnesium levels (all *p* < 0.05). The pathogenesis was undetermined in 17 (45.9%) patients with SMICH compared to 114 (20.3%) with a single ICH (*p* < 0.001) according to the SMASH-U classification. Regarding CSVD markers, patients with SMICH had a higher CMB burden, BG EPVS, and WMH than those with single ICH (all *p* < 0.05).

After backward elimination strategy, age, previous ICH, total WMH score, serum magnesium levels, and deep CMB were retained in the final multivariate logistic regression analysis model. SMICH was associated with the presence of previous ICH (odds ratio [OR] 3.38, 95% confidence interval [CI] 1.31–8.05, *p* = 0.008), higher WMH score(OR 1.27, 95% CI 1.04–1.57, *p* = 0.021), deep CMB burdens (OR 1.06, 95% CI 1.00–1.12, *p* = 0.040), and lower serum magnesium levels (OR 0.01, 95% CI 0.00–0.25, *p* = 0.007).

### Undetermined SMICH group compared to CAA and HTN-ICH groups

In the SMICH group, 17 patients had undetermined etiology, of which one had strictly multi-deep ICH lesions with unknown pre-ICH hypertension, and the rest had mixed lobar and deep/infratentorial hemorrhage. We compared the baseline characteristics of the undetermined-SMICH group with those of the CAA and HTN-ICH groups (Table 2). Compared to patients with CAA-ICH, patients with undetermined-SMICH had more severe deep CMBs (*p *< 0.001) and a high-degree BG-EPVS (*p* = 0.005); however, they were less like to have a high-degree CSO-EPVS (*p* = 0.013). Multiple logistic regression analysis revealed that undetermined-SMICH was independently associated with a severe burden of deep microbleeds, while patients with CAA-ICH was associated with a high-degree of CSO-EPVS (OR 0.02, 95%CI 0.00–0.32, *p* = 0.041; Table 3).

**Table 2:** Comparison of baseline demographics, clinical, and neuroimaging characteristics between patients with undetermined SMICH and those with cerebral amyloid angiopathy ICH and hypertensive angiopathy ICH.

	Undetermined SMICH (*n* = 17)	CAA-ICH (*n* = 85)	*Value of p* ^1^	HTN-ICH (*n* = 382)	*Value of p* ^2^
**Age at enrollment, y**	63 (56–75)	70 (65–79)	0.062	60 (51–69)	0.164
**Male sex**	14 (82.4%)	52 (61.2%)	0.095	246 (64.4%)	0.128
**Hypertension**	11 (64.7%)	48 (56.5%)	0.530	377 (98.7%)	<0.001**
**Diabetes mellitus**	4 (23.5%)	10 (11.8%)	0.244	73 (19.1%)	0.752
**Previous ICH**	4 (23.5%)	7 (8.2%)	0.084	30 (7.9%)	0.047*
**Antiplatelet**	3 (17.7%)	7 (8.2%)	0.364	35 (9.2%)	0.213
**Anticoagulation**	0 (0.0%)	1 (1.2%)	1.000	1 (0.3%)	1.000
**Statin**	2 (11.8%)	3 (3.5%)	0.193	22 (5.8%)	0.272
**APTT, s**	35.10 (34.10–36.30)	34.40 (32.00–38.30)	0.533	34.20 (31.70–36.90)	0.203
**INR**	1.00 (0.99–1.03)	1.02 (0.99–1.08)	0.177	0.99 (0.96–1.04)	0.529
**Creatine, umol/L**	60 (51–76)	60 (51–71)	0.881	61 (51–73)	0.784
**ICH volume, mL**	23.2 (2.7–30.5)	19.4 (9.0–37.2)	0.453	7.0 (2.7–13.6)	0.018*
**Presence of IVH**	8 (47.1%)	22 (25.9%)	0.080	124 (32.5%)	0.211
**Lobar CMB count**	4 (2–7)	2 (0–6)	0.173	0 (0–2)	<0.001**
**Deep CMB count**	6 (2–9)	0 (0–2)	<0.001**	1 (0–4)	0.002*
**Total WMH burden(Fazekas score)**	4 (2–6)	3 (2–5)	0.231	3 (1–4)	0.082
**CSO EPVS high degree (score > 20)**	1 (5.9%)	31 (36.5%)	0.013*	31 (8.1%)	1.000
**BG EPVS high degree (score > 20)**	7 (41.2%)	9 (10.6%)	0.005	71 (18.6%)	0.030
**Lacunes ≥ 1**	14 (82.4%)	58 (68.2%)	0.244	254 (66.5%)	0.173
**Presence of cSS**	4 (23.5%)	36 (42.4%)	0.147	51 (13.4%)	0.271

Data are median (interquartile range) or n (%). Abbreviations: SMICH, simultaneous intracerebral hemorrhages; CAA, cerebral amyloid angiopathy; HTN, hypertension; ICH, intracerebral hemorrhage; APTT, activated partial thromboplastin time; INR, international normalized ratio; IVH, intraventricular hemorrhage; CMB, cerebral microbleed; WMH, white matter hyperintensities; CSO, centrum semiovale; BG, basal ganglia; EPVS, enlarged perivascular spaces; cSS, cortical superficial siderosis. The value of *p*^1^ refer to comparison between patients with undetermined SMICH and CAA-ICH. The value of *p*^2^ refer to comparison between patients with undetermined SMICH and HTN-ICH. *p < 0.05. **p < 0.001.

**Table 3:** Multivariable logistic regression models for factors associated with undetermined SMICH when compared with CAA-ICH and HTN-ICH.

	Compared with CAA-ICH			Compared with HTN-ICH		
	OR	95% CI	*Value of p*	OR	95% CI	*Value of p*
*Model 1*						
Age at enrollment, y	0.95	0.89–1.01	0.083	1.02	0.97–1.06	0.507
Deep CMB count	1.18	1.04–1.37	0.021	1.07	1.00–1.13	0.030
BG EPVS high degree (score > 20)	3.50	0.77–14.9	0.091	2.00	0.61–6.24	0.235
*Model 2*						
Age at enrollment, y	0.96	0.90–1.02	0.205	1.02	0.98–1.07	0.236
Deep CMB count	1.30	1.14–1.57	0.001	1.08	1.02–1.15	0.009
CSO EPVS high degree (score > 20)	0.02	0.00–0.32	0.041	0.53	0.03–3.01	0.562

Abbreviations: CAA, cerebral amyloid angiopathy; HTN, hypertension; ICH, intracerebral hemorrhage; CMB, cerebral microbleed; CSO, centrum semiovale; BG, basal ganglia; EPVS, enlarged perivascular spaces; OR, odds ratio, CI, confidence interval.

Compared to patients with HTN-ICH, patients with undetermined-SMICH had a higher frequency of previous ICH, a more severe burden of CMBs, and a high-degree of BG-EPVS (all *p* < 0.05). The total WMH burden was likely more severe in the undetermined-SMICH group (*p* = 0.082). The final multivariate logistic regression analysis model revealed that undetermined-SMICH was consistently associated with a severe deep CMB (Table 3).

### 90-day functional outcome of SMICH

The 90-day mRS data were available for 571 patients (95.5%). Twenty-seven patients with missing outcome data had single ICH and were excluded from the analysis. In the SMICH group, there were 19 patients with 90-day mRS ≥ 3: 7(46.7%) of 15 patients with HTN-SMICH, 4(80.0%) of 5 patients with CAA-SMICH, and 8(47.1%) of 17 patients with undetermined-SMICH.

**Figure 3:**
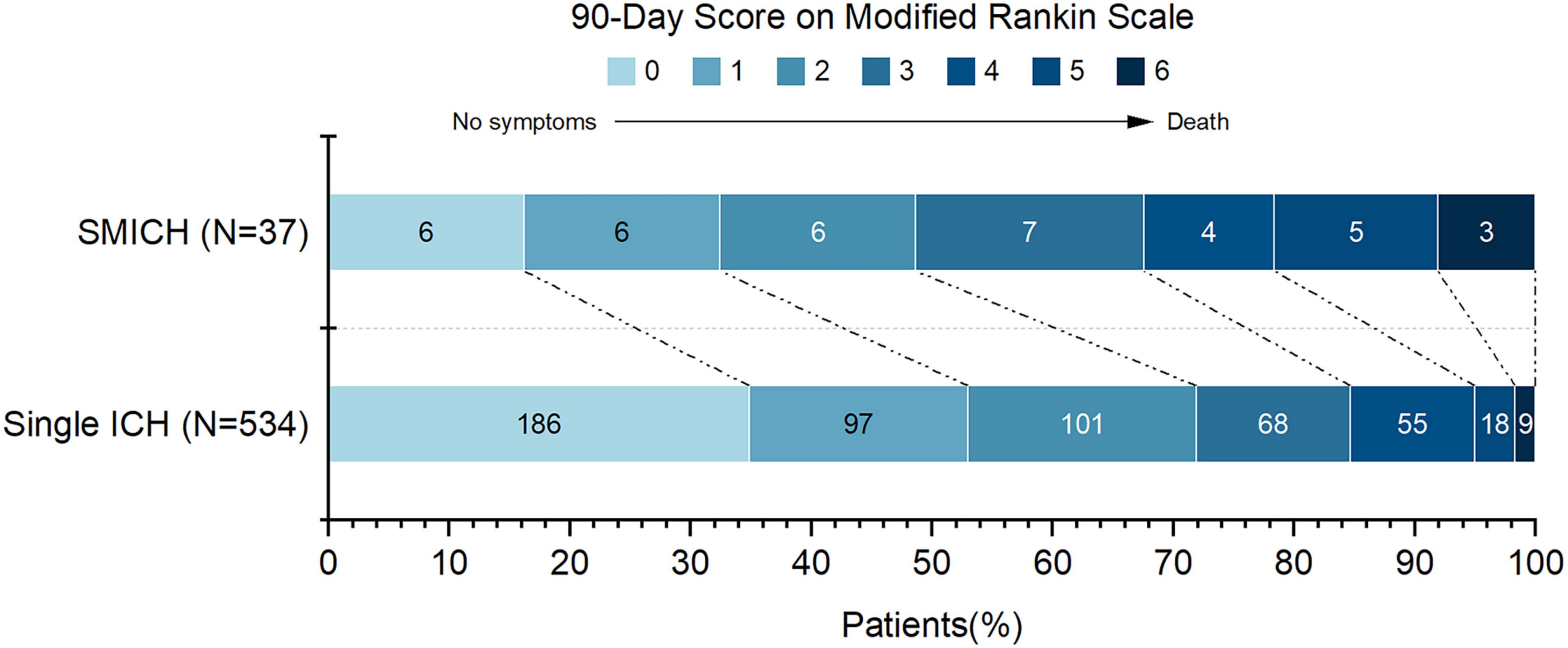
90-Day functional outcomes (modified Rankin Scale score) among patients grouped by simultaneous multiple intracerebral hemorrhage.

Figure 3 illustrates the mRS distribution of patients grouped by SMICH. There was a significant association with a higher frequency of mRS 3–6 in the SMICH group compared to patients with single ICH (51.4% vs. 29.1%, *p* = 0.004). We performed a binary logistic regression analysis for factors associated with a 90-day mRS score ≥ 3. SMICH (OR 2.23, 95%CI 1.03–4.76, *p* = 0.038) was an independent prognostic factor in patients with primary ICH after adjusting for age, baseline GCS score, ICH volume, infratentorial location, and the presence of IVH (Table 4).

**Table 4:** Multivariable logistic regression model for factors associated with 90 day mRS ≥ 3.

	Total Cohort (*n* = 571)^a^			Propensity score matching (*n* = 105)		
	OR	95% CI	*p*-value	OR	95% CI	*value of p*
**Age**	1.04	1.02–1.06	<0.001**	1.04	1.00–1.08	0.041*
**SMICH**	2.23	1.03–4.76	0.038*	3.14	1.09–9.56	0.037*
**GCS**	0.78	0.71–0.86	<0.001**	0.66	0.51–0.81	<0.001**
**ICH volume**	1.03	1.01–1.04	0.001**	1.02	0.99–1.05	0.188
**Presence of IVH**	1.39	0.91–2.11	0.124	1.42	0.50–3.98	0.503
**Infratentorial location**	0.57	0.29–1.07	0.090	1.57	0.24–8.48	0.612

Abbreviations: mRS, modified Ranking Scale; SMICH, simultaneous intracerebral hemorrhages; GCS, glasgow coma scale; IVH, intraventricular hemorrhage; OR, odds ratio, CI, confidence interval. ^a^ Twenty-seven patients with missing mRS data were excluded from the analysis. *p < 0.05; **p < 0.001

Propensity score matching resulted in 105 patients with balanced baseline characteristics (Supplementary Table 2). Approximately 20% of unmatched patients were excluded because no appropriate single ICH cases could be identified within the specified caliper width. Logistic regression after PSM showed that SMICH remained significantly associated with 90-day unfavorable outcome (OR 3.14, 95%CI 1.09–9.56, *p* = 0.037, Table 4).

## Discussion

Our study revealed that SMICH accounted for 6.2% of patients with primary ICH, and was associated with a higher burden of deep CMBs and total WMH. SMICH was an independent predictor of the 90-day unfavorable outcome of ICH. According to the SMASH-U classification, hypertensive angiopathy and cerebral amyloid angiopathy accounted for 54.1% of all the patients with SMICH, whereas 17 of 37(45.9%) patients remained undetermined. Comparing this subgroup to patients with CAA-ICH and HTN-ICH, we observed a consistent independent association between a high deep CMB burden and undetermined-SMICH, whereas patients with CAA-ICH were associated with a high-degree of CSO-EPVS.

The prevalence of SMICH has not been extensively studied. Published data in the past decade revealed an incidence of 3.6–5.9% (Stemer et al., 2010; Yeh et al., 2014; Chen et al., 2016; Wu et al., 2017). Our study provided further evidence that SMICH was not uncommon among primary ICH patients. However, the pathogenesis of primary SMICH differs between studies. According to the SMASH-U classification, 40.5% of the SMICH were HTN-related and 13.5% had CAA. This finding is similar to that of a report by Stemer et al. (2010) where hypertension accounted for 37.9% and CAA for 10.3%. Conversely, analyses by Chen et al. (2016) and Wu et al. (2017) who also classified SMICH patients using the SMASH-U classification system, found a higher percentage of CAA-SMICH than HTN. MRI has emerged as the most useful noninvasive method to unravel the underlying microangiopathies associated with primary ICH; therefore, we undertook an exploratory analysis of the associations between neuroimaging markers of SVD and SMICH. We observed that SMICH was independently associated with a higher burden of deep CMBs and total WMH. Considering the high proportion of patients with undetermined-etiology, we further compared this subgroup with CAA-ICH and HTN-ICH individually. Our results revealed that patients with undetermined-SMICH presented a higher burden of deep CMB with a lower CSO EPVS than those with CAA-ICH. Moreover, undetermined-SMICH inclined to have a greater presence of severe BG EPVS. Based on existing evidence, BG EPVS and deep CMB are associated with HTN-SVD, whereas CSO EPVS are associated with CAA (Greenberg et al., 2009; Charidimou et al., 2013, 2017; van Veluw et al., 2022). Thus, severe hypertensive arteriosclerosis may be the primary denominator of SMICH patients in our study. This is also suggested by our finding that a higher burden of CMB counts while a lower HTN prevalence in undetermined-SMICH group when compared to HTN-ICH. It is likely that some patients were probably unaware of their hypertension and did not adopt interventions, leading to extensive degenerative changes in the SVD caused by longstanding hypertension.

Our study parallels recent studies that observed the mixed-location of lobar and deep intracerebral hemorrhage (ICH)/microbleeds as mostly driven by vascular risk factors to HTN-ICH (Pasi et al., 2018; Tsai et al., 2019). It has been shown that hypertension could promote hemorrhage/microbleeds in lobar locations (Broderick et al., 1993; Vernooij et al., 2008; Kim et al., 2016; Rodrigues et al., 2018). Based on pathology studies, hypertension-related changes also affected leptomeningeal and cortical vessels (Vonsattel et al., 1991; Wakai et al., 1992; Broderick et al., 1993). Results from a recent autopsy study supported this view, revealing that 39% of patients with lobar ICH had moderate or severe arteriolosclerosis alone (Rodrigues et al., 2018). Likewise, in our group, a patient with concomitant lobar and deep location ICH presented no amyloid load in amyloid PET images. CAA and hypertension frequently occur with age, therefore, we could not exempt that the different degrees of CAA co-existing in some patients. Cortical superficial siderosis, a specific hemorrhagic marker of CAA, was observed in 105 (17.6%) patients with SMICH: 97 (17.3%) of 561 patients with single-ICH and 8 (21.6%) of 37 patients with SMICH. Well-designed multicenter prospective studies with detailed vascular risk factors, blood pressure information, and systematic amyloid PET imaging are required to explore the underlying vasculopathy of SMICH.

This study derived another point of interest that low serum magnesium was independently associated with SMICH. In observational studies, low serum magnesium had been associated with larger baseline hematoma volumes, larger final hematoma volumes, and hematoma growth (Liotta et al., 2017; Goyal et al., 2018). Emerging *in vitro* and *in vivo* data support a mechanistic role for magnesium in coagulation, platelet aggregation, and hemostasis (Chang et al., 2019). To synthesize a cohesive pathophysiology, we hypothesized that impaired hemostasis might exert a synergistic effect in patients with extensive CMB burden, provoking the rupture of multiple diseased and vulnerable vessels. However, a clinical trial refuted that magnesium sulfate did not reduce hematoma expansion in patients with acute ICH (Naidech et al., 2022). This question requires further study.

Our study had several clinical implications. Based on our observations, there was an independent correlation between SMICH and the 90-day unfavorable outcomes after adjusting for traditional effectors of prognosis. Consistent with hypertensive arteriopathy accounting for a greater proportion of ICH in the Asian populations, (Chen et al., 2010) our study provides further evidence and implies the necessity of individualized strict blood pressure control. Moreover, it might be worth trying to adopt rehabilitation exercise as soon as possible to improve neurological function in patients with SMICH.

The strengths of our study included its homogeneous patient population with strict inclusion and exclusion criteria, detailed neuroradiological data, and standardized 90-day outcome assessments. In addition, we analyzed hematoma volume using semi-automatic volumetric tools rather than ABC/2 methods, increasing our result accuracy. Nevertheless, some limitations need to be cautious. First, our hospital is a tertiary institution, and selection bias likely occurred, including a higher proportion of patients with SMICH. Second, we prospectively collected neuroimaging and prognostic data using a standard protocol; however, our study had a retrospective design. Therefore, the risk of selection and information bias was inevitable. Third, the relatively small sample size could have restricted statistical validity. Fourth, there was a possibility of missing smaller simultaneous hemorrhages due to the 5 mm image slices of CT. However, all images were analyzed by trained investigators and inter-rater reliability was excellent (kappa = 0.82). Finally, we only recruited patients with MRI images, which could have introduced bias as MRI performed in more stable patients; in fact, patients without MRI presented with more severe neurological deficits and larger hematoma volume in our cohort (Supplementary Table 1).

In conclusion, SMICH was relatively common and was observed in approximately 6.2% of patients with primary ICH. Our results suggest that most patients with primary SMICH harbor hypertensive-SVD as principle angiopathy. Patients with SMICH are at a high risk of poor functional outcomes. Further studies are required to validate these findings and elucidate the etiology underlying primary SMICH.

## Data availability statement

The raw data supporting the conclusions of this article will be made available by the authors, without undue reservation.

## Ethics statement

The studies involving human participants were reviewed and approved by the Human Ethics Committee Board of the Second Affiliated Hospital of Zhejiang University School of Medicine. The patients/participants provided their written informed consent to participate in this study.

## Author contributions

FG and LT have full access to all data in the study and take responsibility for the integrity of the data and the accuracy of data analysis and reviewed, edited, and approved the final version of the manuscript. JL and DS equally contributed to the study and were involved in data analysis and manuscript writing. JL, LT, and FG designed the study. YZ, YJ, LJ, and XY collected the data. All authors contributed to the article and approved the submitted version.

## Funding

This study was funded by the National Natural Science Foundation of China (NSFC; 81471168), the National Natural Science Foundation of China (NSFC; 81971155), the Science and Technology Action Plan for Major Diseases Prevention and Control in China (2017ZX-01S-006S3), and the Science and Technology Department of Zhejiang Province (2022KY174).

## Conflict of interest

The authors declare that the research was conducted in the absence of any commercial or financial relationships that could be construed as a potential conflict of interest.

## Publisher’s note

All claims expressed in this article are solely those of the authors and do not necessarily represent those of their affiliated organizations, or those of the publisher, the editors and the reviewers. Any product that may be evaluated in this article, or claim that may be made by its manufacturer, is not guaranteed or endorsed by the publisher.

## Footnotes

^1^ www.itksnap.org

^2^ https://www.R-project.org/
