# Supplementary material for: Underlying microangiopathy and functional outcome of simultaneous multiple intracerebral hemorrhage
Source: Front Aging Neurosci. 2022 Nov 8;14:1000573. doi: 10.3389/fnagi.2022.1000573 (PMC9679501; doi:10.3389/fnagi.2022.1000573)
Supplement: Supplementary file 1 [file Table_1.DOCX]

**SUPPLEMENTARY MATERIALS**

**Underlying microangiopathy and functional outcome of simultaneous multiple intracerebral hemorrhage**

Jiawen Li, MD^1†^, Dan Shen, MD^1†^, Yanli Zhou, MD^1^, Yujia Jin, MD^1^, Luhang Jin, MD^1^, Xianghua Ye, MD^1^, Lusha Tong, MD, PhD^1*^, Feng Gao, MD, PhD^1*^

^1^Department of Neurology, The Second Affiliated Hospital, Zhejiang University School of Medicine, Hangzhou 310009, Zhejiang, China

^†^Jiawen Li and Dan Shen contributed equally to the present work.

***Correspondence author:** Feng Gao, MD, PhD, and Lusha Tong, MD, PhD, Department of Neurology, The Second Affiliated Hospital, Zhejiang University School of Medicine, 88 Jiefang Rd., Hangzhou, Zhejiang 310009, China. E-mail: [2202012@zju.edu.cn](mailto:2202012@zju.edu.cn) (Feng Gao) and [2310040@zju.edu.cn](mailto:2310040@zju.edu.cn) (Lusha Tong).

Supplementary table 1. Comparison of baseline demographics, clinical, and hematoma characteristics between patients with and without MRI

|  | Without MRI  (n = 282) | With MRI  (n = 598) | *p*-value |
| --- | --- | --- | --- |
| Age at enrollment, y | 65 (55–74) | 62 (52–70) | <0.001^**^ |
| Male sex | 188.0 (66.7%) | 392.0 (65.6%) | 0.745 |
| Onset to emergency, h | 5.0 (3.0–13.0) | 6.0 (3.0–18.0) | 0.182 |
| GCS | 14 (11–15) | 15 (13–15) | <0.001^**^ |
| NIHSS | 8 (3–14) | 4 (2–10) | <0.001^**^ |
| Hypertension | 217 (77.0%) | 453 (75.8%) | 0.697 |
| Diabetes mellitus | 58 (20.6%) | 99 (16.6%) | 0.147 |
| Previous ICH | 25 (8.9%) | 45 (7.5%) | 0.493 |
| Antiplatelet | 30 (10.6%) | 51 (8.5%) | 0.312 |
| Anticoagulation | 10 (3.6%) | 2 (0.3%) | <0.001^**^ |
| Statin | 22 (7.8%) | 32 (5.4%) | 0.158 |
| ICH volume, mL | 10.5 (4.3–24.0) | 9.0 (3.2–17.7) | 0.004^*^ |
| Presence of IVH | 100 (35.5%) | 184 (30.8%) | 0.165 |
| ICH etiology |  |  |  |
| Amyloid angiopathy | 50 (17.7%) | 85 (14.2%) | 0.177 |
| Hypertensive angiopathy | 179 (63.5%) | 382 (63.9%) | 0.907 |
| Undetermined | 53 (18.8%) | 131 (21.9%) | 0.289 |

Data are median (interquartile range) or n (%).

Abbreviations: GCS = glasgow coma scale; NIHSS = national institute of health stroke scale; ICH = intracerebral hemorrhage; IVH = intraventricular hemorrhage.

* p < 0.05, ** p < 0.001

Supplementary table 2. Baseline differences between SMICH and single ICH patients after propensity score matching

|  | Single ICH  (n = 76) | SMICH  (n=29) ^a^ | *p*-value |
| --- | --- | --- | --- |
| Age at enrollment, y | 65 (51, 73) | 63 (54, 75) | 0.505 |
| Male sex | 49 (64.5%) | 18 (62.1%) | 0.819 |
| Onset to emergency, h | 6 (4, 17) | 7 (3, 24) | 0.777 |
| GCS | 15 (13, 15) | 15 (12, 15) | 0.808 |
| NIHSS | 5 (2, 10) | 8 (4, 10) | 0.087 |
| Hypertension | 65 (85.5%) | 22 (75.9%) | 0.257 |
| Diabetes mellitus | 12 (15.8%) | 4 (13.8%) | 1.000 |
| Previous ICH | 14 (18.4%) | 7 (24.1%) | 0.513 |
| Antiplatelet | 11 (14.5%) | 3 (10.3%) | 0.753 |
| Anticoagulation | 0 | 0 | - |
| Statin | 5 (6.6%) | 2 (6.9%) | 1.000 |
| Tobacco use | 22 (29.0%) | 9 (31.0%) | 0.834 |
| Serum Calcium, mmol/L | 2.24 (2.18, 2.35) | 2.30 (2.21, 2.34) | 0.244 |
| Serum Magnesium, mmol/L | 0.85 (0.80, 0.91) | 0.81 (0.77, 0.88) | 0.164 |
| APTT, s | 34.45 (32.08, 36.55) | 35.10 (32.60, 37.60) | 0.346 |
| INR | 1.01 (0.96, 1.06) | 1.00 (0.96, 1.07) | 0.816 |
| Creatine, umol/L | 64 (57, 76) | 58 (48, 66) | 0.180 |
| ICH volume, mL | 15.9 (8.6, 26.6) | 14.8 (7.1, 30.2) | 0.866 |
| Presence of IVH | 25 (32.9%) | 14 (48.3%) | 0.145 |
| ICH etiology |  |  |  |
| Amyloid angiopathy | 13 (17.1%) | 5 (17.2%) | 1.000 |
| Hypertensive angiopathy | 45 (59.2%) | 15 (51.7%) | 0.488 |
| Undetermined | 18 (23.7%) | 9 (31.0%) | 0.441 |

Data are median (interquartile range) or n (%).

Abbreviations: SMICH = simultaneous intracerebral hemorrhages; GCS = glasgow coma scale; NIHSS = national institute of health stroke scale; APTT, activated partial thromboplastin time; INR = international normalized ratio; IVH = intraventricular hemorrhage.

^a^ 8 unmatched patients were excluded because no appropriate single ICH cases could be identified within the specified caliper width.
